# Development and verification of machine learning model based on anogenital distance, penoscrotal distance, and 2D:4D finger ratio before puberty to predict hypospadias classification

**DOI:** 10.3389/fped.2024.1297642

**Published:** 2024-04-30

**Authors:** Zirong He, Bo Yang, Yunman Tang, Xuejun Wang

**Affiliations:** Department of Pediatric Surgery of Children’s Medical Center, Sichuan Provincial People’s Hospital, School of Medicine, University of Electronic Science and Technology of China, Chengdu, China

**Keywords:** machine learning, hypospadias, anogenital distance, penoscrotal distance, 2D:4D finger ratio

## Abstract

**Objectives:**

To describe the anatomical abnormalities of hypospadias before puberty using current commonly used anthropometric index data and predict postoperative diagnostic classification.

**Methods:**

Children with hypospadias before puberty who were initially treated at Sichuan Provincial People's Hospital from April 2021 to September 2022 were selected. We recorded their preoperative penoscrotal distance, anogenital distance, 2D:4D finger ratio, and postoperative hypospadias classification. The receiver operating character curve was used for univariate analysis of the diagnostic predictive value of each index for hypospadias classification in the training set. Binary logistic regression, random forest, and support vector machine models were constructed. In addition, we also prospectively collected data from October 2022 to September 2023 as a test set to verify the constructed machine learning models.

**Results:**

This study included 389 cases, with 50 distal, 167 midshaft, and 172 proximal cases. In the validation set, the sensitivity of the binary LR, RF, and SVM was 17%, 17% and 0% for identifying the distal type, 61%, 55% and 64% for identifying the midshaft type, and 56%, 60% and 48% for identifying the proximal type, respectively. The sensitivity of the three-classification RF and SVM models was 17% and 17% for distal type, 64% and 73% for midshaft type, 60% and 60% for proximal type, respectively. In the Testing set, the sensitivity of the binary LR, RF and SVM was 6%, 0% and 0% for identifying the distal type, 64%, 55% and 66% for identifying the midshaft type, and 48%, 62% and 39% for identifying the proximal type, respectively. The sensitivity of the three-classification RF and SVM models was 12% and 0% for distal type, 57% and 77% for midshaft type, and 65% and 53% for proximal type, respectively. Compared with binary classification models, the sensitivity of the three-classification models for distal type was not improved.

**Conclusion:**

Anogenital distance and penoscrotal distance have a favorable predictive value for midshaft and proximal hypospadias, among which AGD2, with higher test efficiency and stability, is recommended as the preferred anogenital distance indicator. The 2D:4D finger ratio (RadioL, RadioR) has little predictive value for hypospadias classification.

## 1 Background

Hypospadias is a common congenital malformation of the male external genitalia, with an increasing incidence (1). The pathogenesis is related to multiple factors such as failure of urethral fold fusion during the 11th–16th weeks of embryogenesis (2, 3) and insensitivity to androgens, insufficient androgen synthesis, and other non-endocrine factors. The main clinical manifestations are penile curvature, abnormal urethral meatus position, and lack of V-shaped foreskin on the ventral side. Duckett determines the classification of hypospadias according to the position of the urethral meatus. With the refined development of hypospadias surgery, attention has been increasingly given to the importance of local anatomical abnormalities in hypospadias classification. The current classification of hypospadias relies mainly on the quality of the urethral plate, the position of the urethral meatus, and the degree of curvature (4–6). However, this can only be determined during surgery.

If the postoperative classification can be described and predicted preoperatively based on local anatomical data, it will be of great significance for surgeons to design a proper surgical plan and preoperatively communicate with family members.

Anogenital distance in rodents and humans is sexually dimorphic, with males being twice as large as females (7, 8), and has been used as a marker of impaired fetal androgen action for decades (7). A shorter anogenital distance can largely predict external genital malformations at birth and is closely related to reproductive disorders in adulthood (9, 10). Studies have reported that children with hypospadias and cryptorchidism have shorter anogenital distances than their peers (11–14), and proximal hypospadias have shorter anogenital distances than other types of hypospadias (15, 16). However, there is currently no international consensus on the measurement of anogenital distance (9, 12, 17). The lack of uniformity in measurement points and methods has become an obstacle to further research and development. Some studies have proposed (18, 19) that the anoscrotal distance also has good predictive value for disease diagnosis. Therefore, we improved the measurement of longitudinal anogenital distance such as anus-anterior/posterior penis distance and anus-anterior/posterior scrotum distance to explore which measurement values have higher classification test efficiency.

Penoscrotal transposition is often associated with hypospadias (20), but currently lacks an accurate definition and is vaguely described as the scrotum partially or completely appearing in front of the penis. Therefore, we improved the measurement of penoscrotal distance and supplemented the transverse data description of penile-scrotal to evaluate its predictive value for hypospadias classification.

Zheng and Cohn (21) found that early embryonic finger development is balanced by androgen and estrogen signaling. That is, increased embryonic androgen activity increases the length of the fourth finger (resulting in a smaller 2D:4D finger ratio), while increased embryonic estrogen activity decreases the length of the fourth finger (resulting in a larger 2D:4D finger ratio). The 2D:4D finger ratio of the right hand is more sensitive to embryonic hormonal regulation than that of the left hand. Previously, children with hypospadias and cryptorchidism were reported to have smaller 2D:4D finger ratios than normal children. Other studies have also found that proximal hypospadias may cause larger 2D:4D finger ratios than distal hypospadias (22, 23). The study aimed to evaluate whether the 2D:4D finger ratio can be used as an endpoint indicator to predict the classification of hypospadias.

This study utilized commonly used anthropometric indicators to digitize the anatomical abnormalities of hypospadias before puberty and predict postoperative diagnostic classification.

## 2 Methods

### 2.1 Study subjects

This study was approved by the Ethics Committee of the Sichuan Academy of Medical Sciences and Sichuan Provincial People's Hospital [Approval No.: LS (Y) 2020-152]. The study subjects were children with hypospadias before puberty (Tanner stage I) who were initially treated at the Sichuan Academy of Medical Sciences & Sichuan Provincial People's Hospital from April 2021 to September 2022. We recorded their height, weight, body mass index (BMI), preoperative penoscrotal distance, anogenital distance, and 2D:4D finger ratio (RadioL, RadioR) and postoperative hypospadias classification. In addition, we also prospectively collected data from October 2022 to September 2023 as a test set to verify the constructed machine learning models.

#### 2.1.1 Inclusion criteria

Tanner stage I, initially treated hypospadias, without perineal surgery, Asian.

#### 2.1.2 Exclusion criteria

When outcome indicators were lacking in studies on machine learning, imputation methods cannot be used to effectively supplement such data. Therefore, patients with missing data on hypospadias type were excluded.

### 2.2 Classification diagnosis criteria

After correction of penile curvature, hypospadias is divided into: distal type (urethral defect reaches the coronal groove or farther, Type 1), midshaft type (urethral defect in the penile body, Type 2), and proximal type (urethral defect at the junction of the penis and scrotum or proximal, Type 3).

### 2.3 Measurement method of modeling variables

Measurement was conducted by two doctors before anesthesia in children for a total of three times and the average was used in order to minimize the difference between different measurers.

#### 2.3.1 Penoscrotal distance measurement

**Figure 1:**
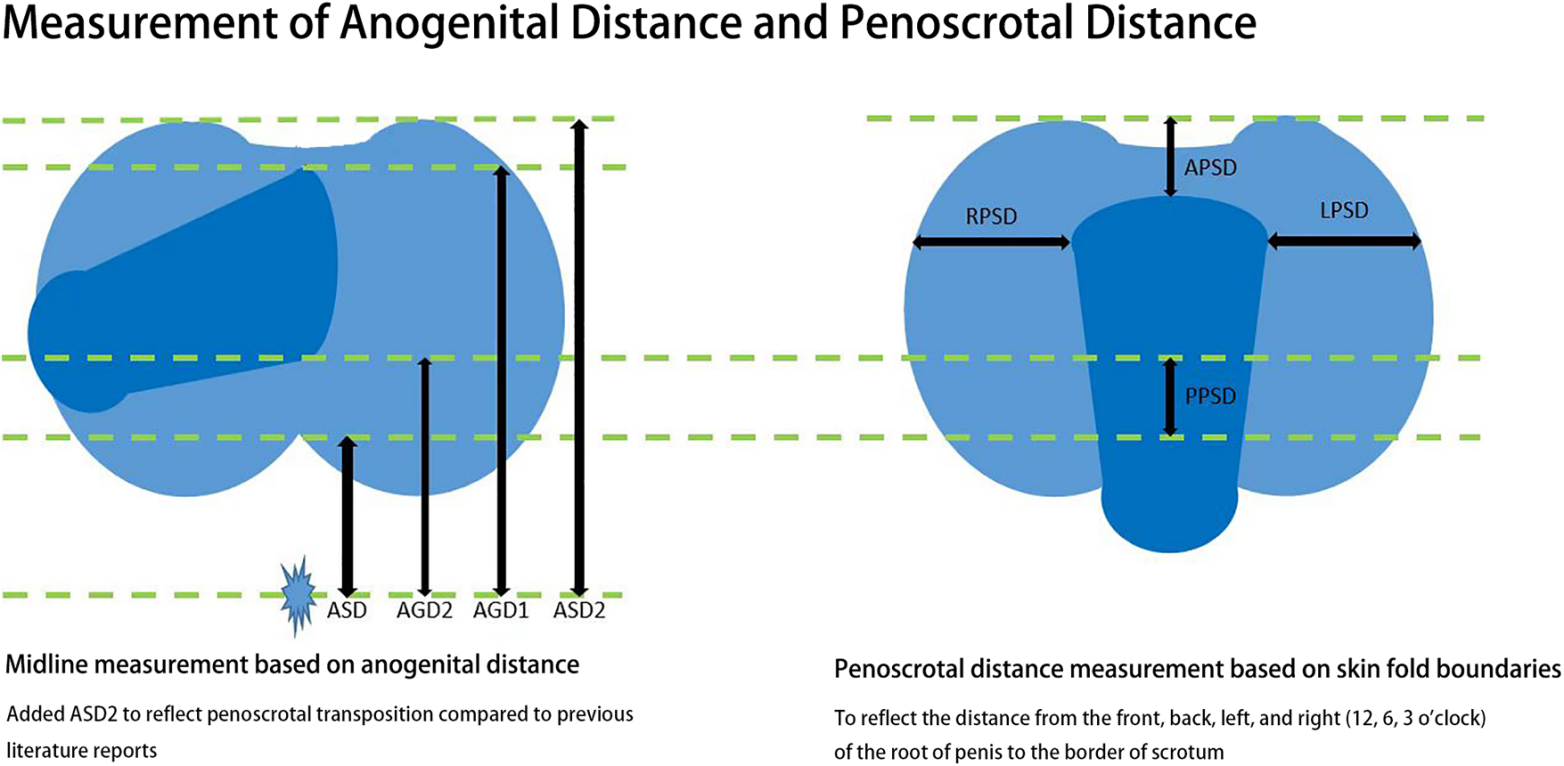
Measurement of anogenital distance and penoscrotal distance.

A steel ruler was used to measure the distance between the 3 o’clock position at the root of the penis and the outer edge of the left scrotum (LPSD), the distance between the 9 o’clock position at the root of the penis and the outer edge of the right scrotum (RPSD), and the distance between the midpoint of the line connecting the upper edges of the bilateral scrotum and the skin fold at the 12 o’clock position at the root of the naturally drooping penis (APSD). The difference between AGD2 and ASD was used as the distance between the 6 o’clock position at the root of the penis and the midpoint of the line connecting the edge of the scrotum (PPSD) (Figure 1).

#### 2.3.2 Anogenital distance

The child was in the frog leg position without traction, the penis was placed in the horizontal position to the right, and the cursor caliper was used to measure the distances on the left midline. Anoscrotal distance (ASD): The distance from the center of anus to the posterior border of scrotum; Anoscrotal distance 2 (ASD2): The distance from the center of anus to the anterior border of scrotum; Anogenital distance 1 (AGD1): The distance from the center of anus to the dorsal midline of the root of penis (12 o’clock position); Anogenital distance 2 (AGD2): The distance from the center of anus to the ventral midline of the root of penis (6 o’clock position) (Figure 1).

#### 2.3.3 The length of the second finger and the fourth finger

A steel ruler was used to measure the distance between the proximal transverse lines of the second/fourth fingers and the fingertips with the fingers spread out flat.

### 2.4 Missing data

To avoid data selection bias, we used multiple interpolation to supplement missing data.

### 2.5 Statistical analysis

In this study, continuous variables were expressed as mean ± standard deviation. Multiple groups were compared using the one-way analysis of variance. LSD-*t*-test was performed for pairwise comparison. The data were randomly divided into the training set and validation set at a ratio of 3:1. Finally, we prospectively collected data from October 2022 to September 2023 as a test set to verify the power of test of these metrical data. In the training set, univariate logistic regression (LR) and receiver operating character (ROC) were applied to analyze the diagnostic value of each indicator for proximal, midshaft and distal types. The optimal Youden's index was adopted to analyze the cut-off values and corresponding sensitivity and specificity of each measurement distance. In addition, we included multiple measurement distances in binary LR, binary random forest (RF) and binary support vector machine (SVM) to diagnose the three types of hypospadias, respectively, in order to explore the diagnostic accuracy of various measurement distances for different types. At the same time, we used three-classification RF and three-classification SVM for multi-class identification of various hypospadias types. These models were validated in the validation set to discuss whether the combination of multiple measurement distances can significantly improve the classification performance compared with a single indicator. *P* < 0.05 indicates a statistically significant difference.

## 3 Results

### 3.1 Differences in indicators between different hypospadias types—one-way analysis of variance

A total of 389 cases were finally enrolled in this study, including 50 cases of distal type (13%), 167 cases of midshaft type (43%) and 172 cases of proximal type (44%). The average age was 33.69 ± 16.64 (months), and the average BMI was15.65 ± 1.81. The results of the one-way analysis of variance showed that except for BMI, RadioR, and RadioL, the remaining measurement indicators (LPSD, PPSD, APSD, RPSD, ASD, AGD2, AGD1, ASD2) were significantly different among the three hypospadias types (*P* < 0.05). There were significant differences in LPSD, PPSD, RPSD, APSD, ASD, AGD2, AGD1, and ASD2 between proximal type and midshaft type, and between proximal type and distal type (Table 1).

**Table 1:** Results of one-way analysis of variance of indicators between different types.

Factors	Type 1 (*n* = 50)	Type 2 (*n* = 167)	Type 3 (*n* = 172)	Overall (*n* = 389)	Statistic	*P*
Month	42.12 ± 22.02	33.87 ± 16.82^c^	31.06 ± 13.68^a^	33.69 ± 16.64	8.92	<0.001
BMI	15.56 ± 1.84	15.64 ± 1.8	15.69 ± 1.92	15.65 ± 1.85	0.10	0.907
LPSD	10.76 ± 3.03	10.23 ± 2.81	13.09 ± 3.67^a^^,^^b^	11.56 ± 3.52	34.58	<0.001
PPSD	39.40 ± 8.59	36.26 ± 6.83	30.90 ± 7.29^a^^,^^b^	34.29 ± 7.93	37.08	<0.001
RPSD	11.00 ± 2.96	10.66 ± 2.99	13.74 ± 3.85^a^^,^^b^	12.07 ± 3.70	37.56	<0.001
APSD	3.06 ± 3.34	3.46 ± 2.81	5.99 ± 3.20^a^^,^^b^	4.53 ± 3.32	35.79	<0.001
RadioL	0.92 ± 0.10	0.93 ± 0.04	0.94 ± 0.03	0.93 ± 0.05	1.32	0.268
RadioR	0.93 ± 0.03	0.94 ± 0.03	0.94 ± 0.03	0.94 ± 0.03	0.12	0.891
ASD	30.66 ± 6.10	28.31 ± 6.39^c^	26.51 ± 5.76^a^^,^^b^	27.81 ± 6.22	9.99	<0.001
AGD2	70.06 ± 9.88	64.56 ± 8.63^c^	57.41 ± 8.85^a^^,^^b^	62.11 ± 9.96	50.31	<0.001
AGD1	83.66 ± 9.49	79.01 ± 8.63^c^	73.48 ± 8.48^a^^,^^b^	77.16 ± 9.37	33.30	<0.001
ASD2	85.42 ± 9.15	80.9 ± 8.45^c^	77.56 ± 7.94^a^^,^^b^	80.01 ± 8.7	18.99	<0.001

^a^ indicates a significant difference between Type 1 and Type 3.

^b^ Indicates a significant difference between Type 2 and Type 3.

^c^ Indicates a significant difference between Type 1 and Type 2.

### 3.2 Differences in modeling variables in training set and validation set

The data were randomized into the training set and validation set, including 195 patients in the training set and 64 patients in the validation set. The level of each indicator was similar between the training set and validation set, but certain differences were observed between the training set and the test set (Table 2).

**Table 2:** Statistical analysis on all indicators in training set and validation set.

Factors	Training set (*n* = 195)	Validation set (*n* = 64)	Testing set (*n* = 130)	Overall (*n* = 389)	Statistic	*P*
Month	36.23 ± 16.12	34.33 ± 16.65	29.55 ± 16.72	33.69 ± 16.64	6.51	0.002
BMI	15.46 ± 1.93	15.50 ± 1.47	16.01 ± 1.87	15.65 ± 1.85	3.74	0.025
LPSD	11.95 ± 3.80	11.48 ± 3.33	11.01 ± 3.09	11.56 ± 3.52	2.87	0.058
PPSD	34.07 ± 8.22	35.94 ± 8.34	33.82 ± 7.19	34.29 ± 7.93	1.69	0.186
RPSD	12.28 ± 3.92	12.02 ± 3.86	11.77 ± 3.27	12.07 ± 3.70	0.76	0.471
APSD	4.32 ± 3.31	3.75 ± 3.34	5.22 ± 3.22	4.53 ± 3.32	5.01	0.007
RadioL	0.94 ± 0.03	0.92 ± 0.08	0.93 ± 0.04	0.93 ± 0.05	4.12	0.017
RadioR	0.94 ± 0.03	0.94 ± 0.03	0.93 ± 0.03	0.94 ± 0.03	2.06	0.129
ASD	28.46 ± 6.20	28.61 ± 7.48	26.45 ± 5.34	27.81 ± 6.22	4.81	0.009
AGD2	62.53 ± 10.28	64.55 ± 11.07	60.28 ± 8.55	62.11 ± 9.96	4.36	0.013
AGD1	77.75 ± 9.50	79.59 ± 10.37	75.08 ± 8.26	77.16 ± 9.37	5.90	0.003
ASD2	80.58 ± 8.68	82.58 ± 9.64	77.88 ± 7.78	80.01 ± 8.70	7.35	0.001
Type					5.35	0.251
Type 1	27 (13.85)	6 (9.38)	17 (13.08)	50 (12.85)		
Type 2	87 (44.61)	33 (51.56)	47 (36.15)	167 (42.93)		
Type 3	81 (41.54)	25 (39.06)	66 (55.77)	172 (44.22)		

### 3.3 Differences in indicators between different hypospadias types—univariate ROC analysis

To clarify which single indicator has better sensitivity and specificity in each type, we analyzed the predictive accuracy of each single indicator for different hypospadias types in the training set and validation set. In the training set, we used univariate LR and receiver operating character (Roc) curves to reflect the classification value of each distance for proximal type, midshaft type and distal type, and the optimal Youden's index to analyze the cut-off values and corresponding sensitivity and specificity of each measurement distance. It was found that the area under the curve of PPSD, APSD, ASD2, AGD2, and AGD1 was all >0.5, better than ASD, with higher sensitivity and specificity. Among them, AGD2 had the best efficiency, while the 2D:4D finger ratio (RadioL, RadioR) had no significant sensitivity and specificity (Table 3).

**Table 3:** Univariate ROC in the training set.

Factors	Type 1 vs. others			Type 2 vs. others			Type 3 vs. others		
	Roc (95% CI)	Sen.	Spe.	Roc (95% CI)	Sen.	Spe.	Roc (95% CI)	Sen.	Spe.
LPSD	0.559 (0.442–0.675)	0.370	0.762	0.694 (0.620–0.768)	0.690	0.639	0.726 (0.655–0.797)	0.691	0.649
PPSD	0.701 (0.592–0.811)	0.481	0.815	0.674 (0.599–0.749)	0.931	0.361	0.776 (0.710–0.842)	0.840	0.579
RPSD	0.576 (0.465–0.687)	0.889	0.292	0.700 (0.624–0.776)	0.678	0.731	0.741 (0.671–0.811)	0.802	0.632
APSD	0.642 (0.518–0.766)	0.481	0.804	0.661 (0.585–0.738)	0.621	0.676	0.734 (0.664–0.805)	0.79	0.632
RadioL	0.604 (0.493–0.714)	0.741	0.488	0.541 (0.459–0.623)	0.241	0.852	0.592 (0.512–0.673)	0.568	0.623
RadioR	0.580 (0.471–0.689)	0.704	0.494	0.506 (0.424–0.588)	0.322	0.704	0.546 (0.463–0.628)	0.568	0.544
ASD	0.594 (0.484–0.705)	0.481	0.69	0.578 (0.498–0.658)	0.494	0.62	0.626 (0.546–0.705)	0.778	0.412
AGD2	0.722 (0.63–0.813)	0.889	0.488	0.691 (0.617–0.766)	0.644	0.676	0.804 (0.741–0.866)	0.728	0.772
AGD1	0.693 (0.597–0.788)	0.852	0.494	0.66 (0.584–0.737)	0.713	0.574	0.758 (0.689–0.827)	0.716	0.746
ASD2	0.668 (0.566–0.77)	0.926	0.375	0.626 (0.547–0.705)	0.632	0.389	0.711 (0.637–0.785)	0.716	0.649

### 3.4 Results of binary classification machine learning

The above observational indicators were included in binary classification machine learning models. In the training set, the sensitivity of the binary LR, RF, and SVM models was 15%, 7% and 11% for identifying the distal type, 62%, 56% and 79% for identifying the midshaft type, and 69%, 64% and 68% for identifying the proximal type, respectively. Meanwhile, their specificity in the training set was 99%, 96% and 100% for identifying the distal type, 69%, 70% and 79% for identifying the midshaft type, and 84%, 81% and 89% for identifying the proximal type, respectively.

In the validation set, the sensitivity of the binary LR, RF and SVM was 17%, 17% and 0% for identifying the distal type, 61%, 55% and 64% for identifying the midshaft type, and 56%, 60% and 48% for identifying the proximal type, respectively. At the same time, the specificity in the validation set was 98%, 93% and 100% for identifying the distal type, 65%, 71% and 74% for identifying the midshaft type, and 85%, 79% and 87% for identifying the proximal type, respectively.

In the Testing set, the sensitivity of the binary LR, RF and SVM was 6%, 0% and 0% for identifying the distal type, 64%, 55% and 66% for identifying the midshaft type, and 48%, 62% and 39% for identifying the proximal type, respectively. At the same time, the specificity in the validation set was 100%, 97% and 100% for identifying the distal type, 69%, 64% and 60% for identifying the midshaft type, and 88%, 78% and 80% for identifying the proximal type, respectively. According to the binary classification machine learning models, each indicator has a favorable diagnostic value for proximal type and midshaft type, but poor sensitivity for distal type diagnosis (Table 4).

**Table 4:** Results of binary LR, RF and SVM analysis.

Model	Classification	Training set				Validation set				Testing set			
		TP	FP	FN	TN	TP	FP	FN	TN	TP	FP	FN	TN
LR	Type 1	4	1	23	167	1	1	5	57	1	0	16	113
	Type 2	54	33	33	75	20	11	13	20	30	26	17	57
	Type 3	56	18	25	96	14	6	11	33	32	8	34	56
RF	Type 1	2	6	25	162	1	4	5	54	0	3	17	110
	Type 2	49	32	38	76	18	9	15	22	26	30	21	53
	Type 3	52	22	29	92	15	8	10	31	41	14	25	50
SVM	Type 1	3	0	24	168	0	0	6	58	0	0	17	113
	Type 2	69	23	18	85	21	8	12	23	31	33	16	50
	Type 3	55	13	26	101	12	5	13	34	26	13	40	51

(1) LR, logic regression; (2) RF, random forest; (3) support vector machine.

### 3.5 Results of three-classification machine learning

The above observational indicators were then included in three-classification machine learning models to explore whether the combination of multiple measurement distances can significantly improve diagnostic performance. In the training set, the sensitivity of the three-classification RF and SVM models was 11% and 11% for distal type, 70% and 86% for midshaft type, and 72% and 80% for proximal type, respectively.

In the validation set, their sensitivity was 17% and 17% for distal type, 64% and 73% for midshaft type, and 60% and 60% for proximal type, respectively.

In the Testing set, their sensitivity was 12% and 0% for distal type, 57% and 77% for midshaft type, and 65% and 53% for proximal type, respectively. Compared with binary classification machine learning, the sensitivity of three-classification models for distal type was not improved (Table 5).

**Table 5:** Results of three-classification LR, RF and SVM analysis.

Type	Training set						Validation set						Testing set					
	RF			SVM			RF			SVM			RF			SVM		
	Type1	Type2	Type3	Type1	Type2	Type3	Type1	Type2	Type3	Type1	Type2	Type3	Type1	Type2	Type3	Type1	Type2	Type3
Type 1	3	20	4	3	20	4	1	5	0	1	4	1	2	13	2	0	14	3
Type 2	4	61	22	0	75	12	3	21	9	0	24	9	5	27	15	0	36	11
Type 3	0	23	58	0	16	65	3	7	15	0	10	15	1	22	43	1	30	35

### 3.6 Ranking of variable importance of three-classification machine learning

**Figure 2:**
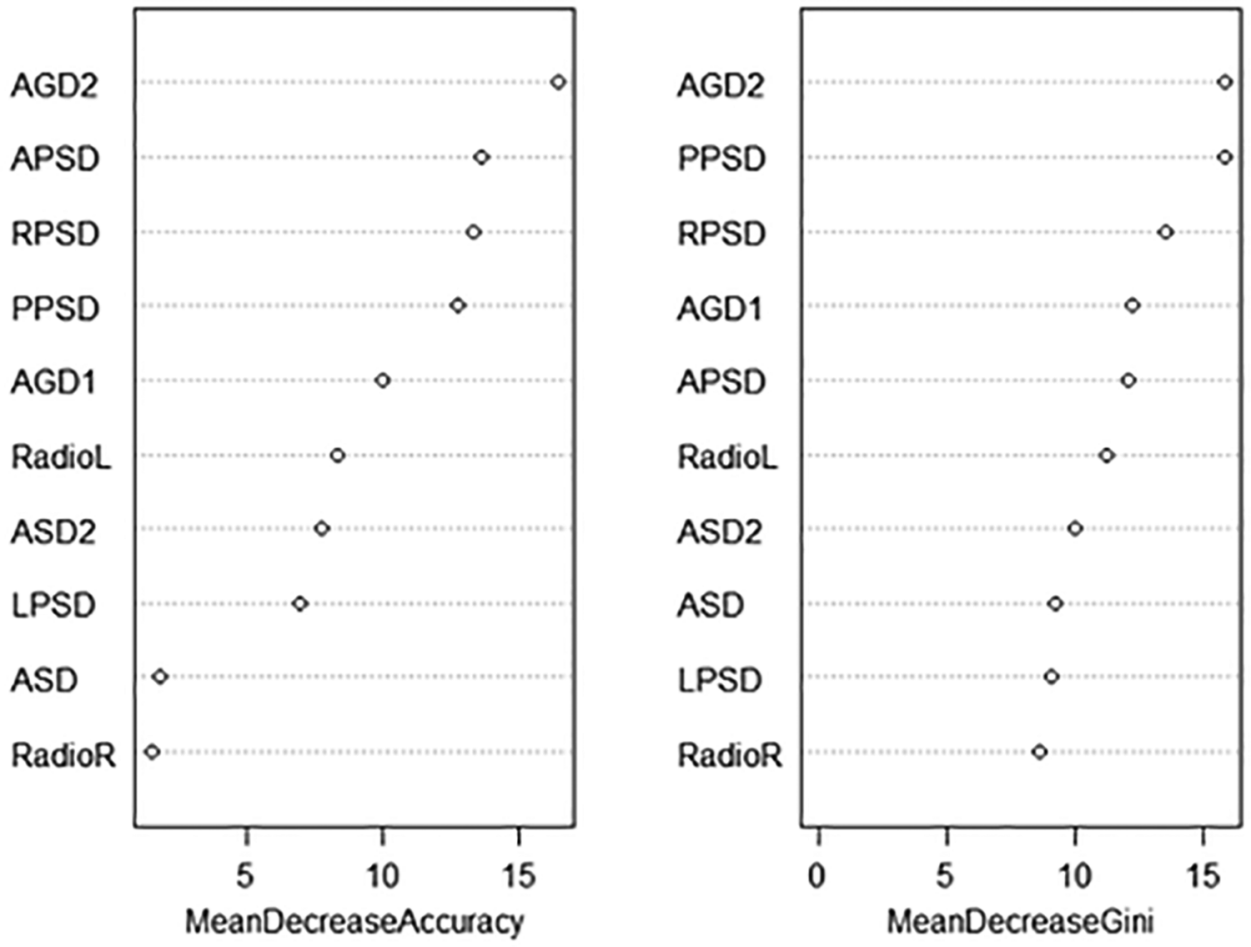
Ranking of variable importance in three-class RF.

In clinical practice, the importance of variables and their contribution to the model need to be considered. Our study found that in the ranking of variables in random forest model, AGD2 made the greatest contribution, while RadioR had the smallest contribution. Some differences were found in the ranking of other variables (Figure 2).

## 4 Discussion

Accurate preoperative classification of hypospadias can guide surgeons in the selection of surgical procedures and improve the effects of surgical treatment. Using high-quality outcome data to convey the benefits (and risks) of surgery to patients and/or family members is also the goal of the informed consent process.

Anogenital distance is determined early in pregnancy and can be used as a biomarker to reflect *in-utero* androgen exposure during early human pregnancy (8–14 weeks) (24). Animal experiments have confirmed that a lack of androgen exposure *in utero* (exposure to estrogen) can lead to shorter anogenital distances (25). A short anogenital distance is associated with hypospadias, cryptorchidism, testicular germ cell tumors, oligospermia/asthenospermia, and other disorders of testicular development (13, 26). Since 2008, anogenital distance has been used as a quantitative biomarker of human fetal exposure to endocrine disruptors (18, 24). However, there is currently no international consensus on the measurement of anogenital distance. Traditional anogenital distance measurements include anus-scrotum posterior distance, anus-penis anterior distance, and anus-penis posterior distance. Existing studies are limited to one or some of these (16, 24, 27) and cannot fully describe the longitudinal anatomy of the penis and scrotum in hypospadias. In clinical practice, we have observed that proximal hypospadias is often associated with anterior displacement of the scrotum. Therefore, in this study, we first proposed to include anus-scrotum anterior distance (ASD2) in the measurement indicators of anogenital distance. Our results show that the closer the urethral meatus is to the perineum, the shorter the ASD2 value, which is consistent with the trend of anus-scrotum posterior distance reported in previous literature (16). However, ASD2 has higher stability than ASD in predicting hypospadias classification. Among all the measured longitudinal indicators of penile-scrotal anatomical abnormalities, AGD2 has the highest predictive value for classification, which is the same as previous literature reports (12, 15). Hence, we recommend AGD2 as the preferred anogenital distance indicator.

Penoscrotal transposition is an anatomical abnormality of penile and scrotal position, but currently, its diagnosis is based primarily on subjective visual description, that is, part or all of the scrotum appears in front of the penis. Normal boys and patients with concealed penis may also have a line connecting the upper edges of the scrotum higher than the dorsal side of the penis due to thicker prepubic fat and poor penile fascia development, but this cannot be diagnosed as penoscrotal transposition. Proximal hypospadias is often associated with penoscrotal transposition (20). The degree of transposition is often related to insufficient masculinization (28, 29). Abbas (30) reported that the scrotal base distance (the distance between the junction of the penis and scrotum and the junction of the perineum and scrotum behind) in children with hypospadias and cryptorchidism can be used as an objective anthropometric indicator and biomarker to evaluate the effects of endocrine disorders in the fetal period on the development of male external genitalia. Therefore, we first proposed LPSD, PPSD, RPSD, and APSD (the distances between the 3 o’clock, 9 o’clock, 6 o’clock and 12 o’clock positions of the penis root and the corresponding edge of the scrotum as indicators of penoscrotal distance) to objectively digitize penoscrotal transposition and analyze its predictive value in hypospadias classification. In this study, the trend of penoscrotal transposition in proximal hypospadias was more obvious than in midshaft and distal hypospadias. The area under the curve of APSD and PPSD (6 and 12 o’clock), reflecting the longitudinal penoscrotal distance, was >0.5, with a certain sensitivity and specificity for classification prediction. However, compared with anogenital distance, it did not show a greater advantage in classification prediction.

The 2D:4D finger ratio exhibits sexual dimorphism. Prenatal androgens affect the development of fingers through androgen receptors. Androgen receptor inactivation will lead to a shorter fourth finger (increased 2D:4D finger ratio). Estrogen receptor inactivation will lead to a longer fourth finger (decreased 2D:4D finger ratio), and the 2D:4D finger ratio of the right hand is more significant than that of the left hand (21). There are many reports on the 2D:4D finger ratio in children with congenital adrenal hyperplasia (31, 32). The 2D:4D finger ratio of 21-hydroxylase deficiency female patients was lower than that of healthy girls but comparable to male controls. The 2D:4D finger ratio of 21-hydroxylase deficiency male patients was significantly lower than that of healthy females and males. Abbo (22) reported that children with cryptorchidism/hypospadias had a significantly lower 2D:4D finger ratio than normal controls. However, photocopies of both hands were used to calculate the finger ratio in that study, and the association between 2D:4D finger ratio and hypospadias classification was not elucidated. O’Kelly (23) reported that children with proximal hypospadias had a higher 2D:4D finger ratio than children with distal hypospadias, and the 2D:4D finger ratio of children with distal hypospadias did not differ from that of controls. In this study, the 2D:4D finger ratio of both hands did not show better predictive diagnostic values in hypospadias classification than anogenital distance and penoscrotal distance. The 2D:4D finger ratio is not currently recommended as a routine measurement indicator to predict hypospadias classification.

## 5 Strengths and limitations

In this paper, the prediction of preoperative local anthropometric indicators for postoperative diagnosis and classification was discussed for the first time, and a machine learning model was also constructed. Our study has revealed that the current anthropometric indicators are of great value for the prediction and diagnosis of midshaft and proximal types. However, our study also has some limitations. First, normal males were not enrolled as a control group for comparison at the same time. Second, the proportions of midshaft and proximal types were relatively high, which differed greatly from the distribution of hypospadias in the population. However, this was caused by the prevalence rate in patients.

## 6 Conclusions

Anogenital distance and penoscrotal distance have good diagnostic predictive values for midshaft and proximal hypospadias. AGD2 has higher test efficiency and stability and is recommended as the preferred anogenital distance indicator. It is also recommended to include ASD2 in the measurement of anogenital distance. The 2D:4D finger ratio (RadioL, RadioR) has little predictive value for classification. Preoperative anthropometric indicators can improve clinical decision making, surgical planning, and parental counseling.

## Data Availability

The original contributions presented in the study are included in the article/Supplementary Material, further inquiries can be directed to the corresponding authors.
